# The quantity-quality tradeoff: a cross-national, longitudinal analysis of national student-faculty ratios in higher education

**DOI:** 10.1007/s10734-020-00621-3

**Published:** 2020-10-29

**Authors:** Elizabeth Buckner, You Zhang

**Affiliations:** University of Toronto, Ontario Institute for Studies in Education, Toronto, Ontario Canada

**Keywords:** National student-faculty ratio, Higher education quality, Massification, Cross-national analysis

## Abstract

This article analyzes cross-national trends in national student-faculty ratios (SFRs) over the past five decades. In descriptive analyses, we find that SFRs have increased globally, driven by particularly large increases in low-income countries. We analyze two cross-national datasets to examine factors associated with national SFRs. We find that national SFRs are positively associated with gross tertiary enrollment rates and particularly so in low-income countries. In contrast, both the female share of faculty and research spending are associated with having lower national SFRs. The findings shed light on how national higher education systems are responding to massification pressures and suggest that differentiating faculty roles is one way that countries curb their rising SFRs as enrollments grow.

## Introduction

One of the fundamental shifts affecting higher education globally has been massification: enrollments in higher education have increased dramatically over the past 50 years in almost every country (Baker 2014; Marginson 2016; Schofer & Meyer 2005; Scott 1995; Tight 2019; Trow 1972, 2007). In light of this expansion, there is the general assumption that massification has resulted in rising average class sizes and increasing student-faculty ratios (SFRs) at both the institutional and national levels (Chang et al. 2015; Hornsby & Osman 2014; Mohamedbhai 2014; Saint 2004). This is a major concern for the field of higher education because increasing numbers of students per faculty are thought to be associated with fewer opportunities for faculty-student interactions and general concerns over the quality of learning.

However, there has been very little systematic analysis of national SFRs in higher education worldwide over time, and as a result, we have little understanding of global trends or factors associated with changing SFRs. Important unresolved questions for the field include the following: have SFRs increased over time? If so, by how much? Have different regions of the world been affected differentially? What factors are associated with changes in SFRs in higher education?

This article brings new cross-national and longitudinal data to analyze national SFRs in higher education over time. Our purposes are twofold: first, we seek to document cross-national and longitudinal trends; secondly, we seek to develop a conceptual framework that explains variation in national SFRs in higher education. SFRs, defined as the total number of students per faculty member, are not the same as average class size, and therefore do not necessarily speak to students’ experiences or the pedagogical issues related to growing class sizes. That said, the SFR is an important indicator of higher education capacity, and we argue, warrants greater attention among scholars of comparative higher education. In particular, the SFR captures the instructional capacity of the system relative to student demand. Moreover, because faculty members are also the research core of the contemporary higher education system, the SFR can be an indicator of a system’s relative focus on research, as compared to teaching.

Relying on data from UNESCO Institute of Statistics (UIS), we examine trends in national SFRs between 1970 and 2018. We find that national SFRs are associated with massification—globally, SFRs have increased markedly since the 1970s. Yet, there is also significant variation across countries: while SFRs have increased dramatically in low-income countries, they have hardly changed in high-income countries. Low-income countries are most affected by enrollment expansion. However, expansion is only part of the story. Regression findings suggest that increases in research spending and the female share of faculty are both associated with having lower SFRs. Interestingly, the female share of faculty is more strongly associated with lower SFRs in high-income and middle-income countries than it is in low-income countries. The results support the idea that role specialization in the professorship between research-active and teaching-oriented faculty may contribute to lowering SFRs at the national level, and yet, also suggests that these relationships vary by national wealth. The findings invite future studies on other factors associated with SFRs, including contract labor.

## Literature review

The topic of class size, particularly in primary and secondary school, has received significant attention in the educational literature, with studies seeking to determine the causal effect of class sizes on student learning (Konstantopoulos & Chung 2009; Konstantopoulos & Li 2012; Watson et al. 2013). At lower levels of schooling, the teacher-pupil ratio is also widely used as an indicator of school quality, despite many critiques (Card & Krueger 1996; Paananen 2020). In international development, organizations such as UNESCO have called for pupil-teacher ratio ceilings of approximately 40:1 (Harber 2014; United Nations Educational Scientific and Cultural Organization UNESCO 2010)

However, there has been less systematic study of SFRs in higher education, particularly in comparative perspective. Country-specific studies indicate that SFRs are associated with institutional characteristics and may be linked to student-level outcomes, although the empirical base for the latter is less clear. For example, studies in the USA have found that SFRs have increased most at institutions that invest in scientific research, suggesting that investments in research result in institution-level trade-offs, including higher SFRs (Ehrenberg et al. 2003; Goenner & Snaith 2004).

Despite a weak empirical base, the SFR is widely used by policymakers and the media as a measure of institutional quality, evidenced by their increasing incorporation into national rankings and policy debates (Schenker-Wicki & Inauen 2012). Schenker-Wicki & Inauen (2012) state that the SFR is “becoming increasingly important to higher education policy” (p. 33). Institutions’ SFRs are frequently used as an indicator of institutional quality, particularly in international and national rankings (Hazelkorn 2015; Schenker-Wicki & Inauen 2012). The US News and World Report uses both average class size (8% total weight) and SFRs (1% total weight) in its national rankings of American colleges and universities. In international rankings, the QS rankings weights SFRs at 20% of total score, while the Times Higher Education ranks the academic staff to student ratio at 4.5% of total score, as part of its indicator of the “Learning Environment.” In some cases, governments have used institutional or department-level SFRs to allocate resources. For example, Switzerland has used the department-level SFR to allocate additional resources to certain university programs (Schenker-Wicki & Inauen 2012).

However, the empirical basis for using the SFR as an indicator of overall quality is thin. Research has found that an institution’s SFR is not necessarily related to student learning outcomes (Toutkoushian & Smart 2001). In fact, a study from the USA found that the institutional SFR is actually positively associated with the five and six-year graduation rates among research-intensive universities (Goenner & Snait 2004). The authors point out that this is unexpected and that the causal mechanism for this finding is unclear.

Moreover, most of this research has been conducted at the institutional level – there has been little study of national SFRs in longitudinal or comparative perspective. Despite this lack of research on SFRs, the national SFR is nonetheless being used in a variety of settings as an indicator of system-wide quality. For example, some ministries use the national SFR in strategic planning, on the assumption that the national SFR is an indicator for aggregate system-capacity or quality (Hayward 2017). The Government of Afghanistan’s education sector plan seeks to maintain a SFR of 25:1 (Hayward 2017). Similarly, in its 2005–2015 strategy paper, the Rectors Conference of the Swiss Universities (CRUS) set a ratio of 60:1 for all bachelor’s degree programs, and at the Master’s level, 40:1 for humanities and social sciences, and 35:1 in technical sciences. Given its incorporation into national planning, it is important to develop a deeper understanding of this indicator and how it varies cross-nationally.

Another important gap in the literature concerns why, when, and how national SFRs change. Throughout the field of higher education development, there is the widespread assumption that SFRs are increasing cross-nationally in response to massification pressures (Saint 2004). For example, Saint (2004) finds that in response to massification, SFRs in Ethiopia roughly doubled between 1995 and 2003. However, in many contexts, massification has been accompanied by significant changes in the academic labor market (Altbach 1999; Finkelstein & Jones 2019). One of the major changes globally since 1970s has been the increasing proportion of faculty members who are women, a result of increased female enrollments in university, among other factors (Wotipka et al. 2018). While scholars recognize that this growth has important implications for student learning and knowledge creation, it may also affect the national SFR, if it reduces the cost of academic labor.

Another change affecting the global academic labor market is the increasing reliance on contract faculty. Neoliberal approaches to higher education and competing policy demands have resulted in fiscal pressures on universities, with many universities increasing contract faculty positions as a response (Altbach et al. 2009; Davies & Bansel 2005; Finkelstein & Jones 2019). For example, Altbach et al. (2009) estimate up to 80% of all faculty in Latin America may be working part-time and that less than half of all new faculty in the USA are on the tenure track. The increasing dependence on contract faculty has contributed to a differentiation of roles, with some faculty focusing on teaching, while others focusing more on research.

In line with this trend of role differentiation, many countries have pursued differentiation within their higher education systems, often by investing more in elite research-intensive institutions with the goal of creating world-class institutions or moving up in global rankings (Carnoy et al. 2013; Chang et al. 2009; Guri-Rosenblit et al. 2007; Mok 2003; Teichler 1998). Comparative data from UNESCO shows that countries around the world have increased spending on research and development (R&D) as a percentage of their GDPs over the past two decades (UNESCO 2020), and have incentivized research productivity and impact (Auranen & Nieminen 2010; Geuna & Martin 2003). An increased focus on research may have implications for national SFRs, as increased spending on R&D, when funneled into the higher education system, may result in more faculty overall, as research-intensive faculty tend to have reduced teaching loads. However, little comparative work has explored how these aspects of the changing labor market are related to national SFRs. In this article, we seek to deepen understanding of the SFR as an indicator of system capacity and to identify factors associated with change.

## Theoretical framework

This article draws on the literature in comparative higher education to hypothesize factors associated with national SFRs over time. The SFR is calculated as the total number of students in the higher education system, divided by the total number of faculty members working in the higher education system. As a relative indicator, the SFR can be affected by changes in either the numerator (i.e., student enrollments) or the denominator (i.e., total faculty). Relative indicators, such as the SFR, can be difficult to analyze, as changes in the overall ratio could result from either a change in the numerator (e.g., fewer students) or a change in the denominator (e.g., more faculty). However, in the case of the SFR, there is a strong justification for examining the ratio, rather than simply the total number of students or total number of faculty in a system. This is because in recent decades, the total number of both students and faculty have generally been increasing in higher education systems (Barakat & Shields 2019; Schofer & Meyer 2005; Trow 2007). Given the fact that both the numerator and denominator have generally increased over time, and substantially so in most countries, we can interpret change in a relatively straightforward manner: an increasing SFR occurs when enrollments increase at a faster rate than increases in total faculty, while a decrease occurs when the total number of faculty increases relative to total student enrollments. Essentially, the SFR sheds light on whether faculty numbers have been “keeping up” with increases in student enrollments. Moreover, in substantive terms, it is the ratio of students per faculty member that higher education scholars are most interested in, as the relative numbers of students per faculty most closely align to the idea of interpersonal interactions between students and faculty. Below, we outline specific factors that likely affect the SFR.

### Total students

Enrollments: We hypothesize that the total number of faculty reflects a functional need for qualified personnel to teach students, and therefore, will be related to the total number of students in higher education.

### Total faculty

Government spending: We also assume that the SFR will reflect government spending on higher education, as more resources funneled into the system means that governments and universities can hire more faculty overall. Prior research on student enrollments in higher education has found that government spending is positively associated with student enrollments, which likely results in additional faculty hiring (Yang & McCall 2014). In contrast, countries that spend fewer revenues on higher education overall may be unable or unwilling to hire faculty at equivalent rates. However, government spending is only one type of funding, and in many countries, private contributions to higher education through tuition and fees are substantial. Unfortunately, reliable cross-national and longitudinal data on private contributions to higher education are not available, so our analysis focuses on government spending.

Labor costs: A significant body of research has found that female faculty are more likely to be younger, concentrated in fields with lower compensation on average, and experience pay discrimination (Finkelstein 2012; Mason & Goulden 2004; Renzulli et al. 2013). They often advance through the ranks at a slower pace and have lower publication productivity. For these reasons, higher proportions of female faculty may represent one form of less costly academic labor, who reduce national SFRs by increasing the overall number of faculty.

A second factor that likely brings down labor costs is the percentage of all faculty who are on contract, as contract labor tends to represent a less costly form of labor (Altbach & Pacheco 2012; Finkelstein 2012; Welch 2012). Unfortunately, we do not have data on the percentage of faculty who are on contracts over time for the many countries in our dataset. We hope that future research will be able to tackle the question with better data on the topic of contingent labor. In our models, we use the female share of all faculty, which is available at the national level starting in 1970s. We think it may be operationalizing the broader concept of labor costs, including contract-faculty, rather than simply gender, but this is an area for future studies to explore.

Research intensity: We hypothesize that research intensity will be associated with a lower SFR, as research-active faculty tend to spend more time on research, and thereby, teach less, requiring more faculty members overall. In their theoretical model of SFRs, Schenker-Wicki & Inauen (2012) argue that SFRs are closely related to how faculty members spend their time, with research-oriented faculty spending significantly less time on teaching. Although their analysis is conducted at the institutional level, the same factors likely matter at the national level—a higher proportion of research-active faculty will likely be negatively associated with the national SFR. To test these hypotheses, we carry out descriptive and multi-level panel regression models to examine factors associated with national SFRs, as discussed below.

## Data, methods, and analysis

### Data

This paper draws on UNESCO Institute of Statistics data. We create two datasets: one with the tertiary pupil-teacher ratio (i.e., SFR) for 166 countries between 1970 and 2018, and a second, richer set of data for 107 countries between 2000 and 2018, when more national correlates are available. These data are reported by national governments to UIS, and as such, definitions may vary somewhat across countries. That said, prior research on comparable indicators has found that definitions tend to be consistent within countries over time (Aksnes et al. 2017). Therefore, by using fixed-effects models, we are able to account for country-specific definitions of who counts as faculty.

Another important limitation to the data concerns its external validity. National ministries and statistical agencies report their data directly to UIS, and in that sense, is officially endorsed. However, UIS does not externally verify reported figures or collect its own data. Therefore, it is possible that UIS data may conflict with other datasets. However, given the lack of comparable data on the topic, UIS remains the best source for cross-national and longitudinal data. We hope that future studies can replicate our analysis as other data become available.

### Variables

The dependent variable in our analysis is the tertiary SFR, defined by UIS as the total number of students per faculty member in tertiary education at the national level. Tertiary education, as defined by UNESCO, includes short-cycle, bachelor’s, master’s, and doctoral degrees or their equivalents.

Independent variables in the 1970–2018 models include gross domestic product (GDP) per capita, spending on tertiary education as a percentage of GDP, gross tertiary enrolment ratio (GTER), the share of female faculty in tertiary education, and country income group. GDP per capita is calculated using the national gross domestic product divided by total population and is used to indicate a country’s overall wealth. Because GDP per capita is not normally distributed, we log it to ensure a normal distribution and improve model fit. Spending on tertiary education as a percentage of GDP is an indicator of a country’s financial investment in tertiary education. Currently, there is no specific indicator for spending on tertiary education as a percentage of GDP in UIS, but there are indicators for both a country’s spending on education as a percentage of its GDP and for spending on tertiary education as a percentage of total public spending on education. We use these two indicators to calculate spending on tertiary education as a percentage of GDP. We use GTER as an indicator of student enrollments. It is calculated as the total number of students enrolled in tertiary education, regardless of age, divided by the total number of individuals who are in the five-year age cohort after the official end of secondary education. The female share of faculty is calculated as the percentage of all faculty in tertiary education who are female. For country income groups, we use World Bank classifications. The World Bank classifies countries into four groups based on their per capita gross national income (GNI): low income (< 1025 USD), lower-middle income (1026–3995 USD), upper-middle income (3996–12,375 USD), and high income (> 12,376 USD).

In the 2000–2018 models, we also add an additional independent variable to operationalize research intensity: spending on research and development (R&D) as a percentage of GDP. Spending on R&D is defined the gross domestic expenditure on research and development as a percentage of GDP. As research is a primary function of faculty in higher education, spending on R&D operationalizes research intensity of faculty labor, and we hypothesize that more research spending will lower the national SFR. Table 1 shows the summary statistics for the 1970–2018 dataset, while Table 2 shows summary statistics for the 2000–2018 dataset.

**Table 1:** Summary statistics for 1970-2018 models (N=3067, 166 Countries)

**Variable **	**N**	**Mean**	**SD**	**Min**	**Max**
SFR	3067	14.78	6.23	4.51	36.16
GDP per capita (logged)	3067	8.41	1.52	5.09	11.61
Tertiary Spending (% of GDP)	3067	0.83	0.49	0.00	4.40
GTER	3067	25.69	24.28	0.01	117.10
Female Faculty (%)	3067	31.74	14.89	1.23	81.98

*Note.* SFR = Student-faculty Ratio; GDP = Gross Domestic Product; GTER = Gross Tertiary Enrollment Ratio

**Table 2:** Summary statistics for 2000-2018 models (N=1218, 107 Countries)

**Variable**	**N**	**Mean**	**SD**	**Min**	**Max**
SFR	1218	15.96	6.09	4.59	35.83
GDP per capita (logged)	1218	8.77	1.50	5.27	11.61
Tertiary Spending (% of GDP)	1218	0.98	0.53	0.00	4.40
GTER	1218	42.42	26.04	0.71	117.10
Female Faculty (%)	1218	39.85	13.23	1.23	81.32
R&D Spending (% of GDP)	1218	0.75	0.86	0.01	4.55

*Note*. SFR = Student-faculty Ratio; GDP = Gross Domestic Product; GTER = Gross Tertiary Enrollment Ratio; R&D = Research and Development.

### Missing data

There are substantial missing values in both datasets. When possible, we use linear interpolation to fill in missing data for all independent variables in the model, except for GDP per capita, which has sufficient observations. If variables still contained a significant number of missing values, we used the country mean to fill in the missing data and included a binary variable indicating whether an observation was previously missing or not (Cohen et al. 1983). We provide the number of missing values filled for all variables (see Tables 5 and 6 in the Appendix).

### Analytic strategy

We first examine how SFRs have changed over time using descriptive analysis. To do this, we graphed SFRs over time and plotted the bivariate relationship between SFR and GTER for different groups of countries. Next, we use fixed-effects panel regression models to examine factors associated with SFRs in both datasets. As the observations are grouped within countries, the unit of analysis is country-year. Using fixed-effects models allows us to see changes within countries. A major assumption of fixed-effects models is that the error terms are correlated with regressors. To test this hypothesis, we conducted a Hausman test and it confirmed that fixed-effect models better suit our data than random effects in both datasets.

Considering the potential for spurious regression if a panel model excludes time effects, we test the need for year-specific fixed effects in STATA. The results showed that year fixed effects are needed in both the 1970–2018 models (*p* < 0.001) and the 2000–2018 model (*p* < 0.05). Considering that our two datasets span nearly 50 years and 20 years, respectively, we include indicator variables for each year in both sets of models. We also use robust standard errors in all models to account for heteroscedasticity.

## Findings

### Global trends

**Fig. 1:**
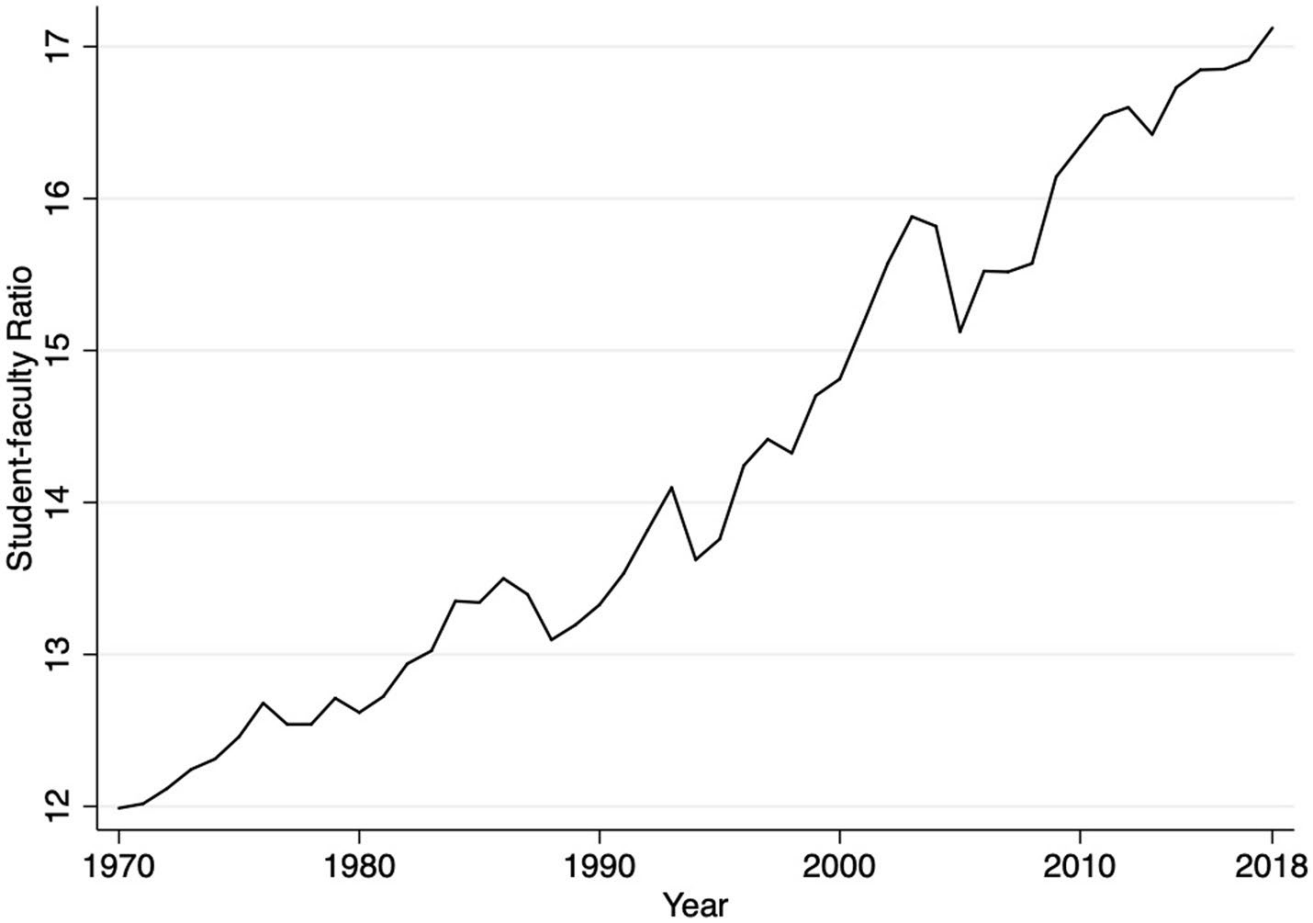
The worldwide student-faculty ratio, 1970–2018

Figure 1 shows the worldwide SFR from 1970 to 2018. There is no indicator for the world’s average SFR; therefore, we calculated the global average by dividing total student enrollment globally by the total number of faculty globally for each year. As shown in Fig. 1, the global SFR has increased rapidly in the past five decades, from roughly 12 in 1970 to over 17 in 2018.

**Fig. 2:**
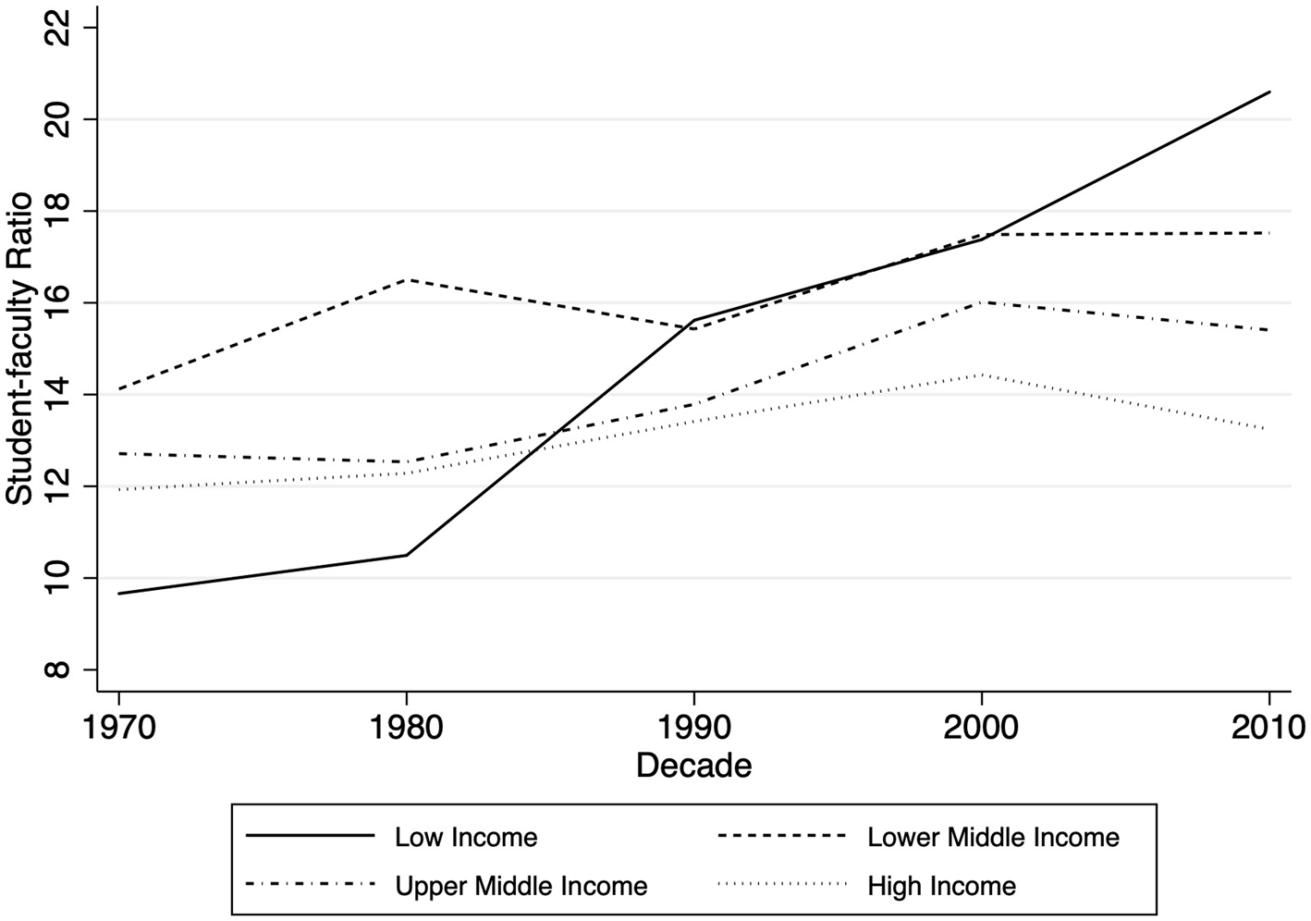
Student-faculty ratios by country income group, 1970–2018

Figure 2 shows SFR trends by income groups, as defined by the World Bank. The figure shows clearly that there are significant differences across income groups. The SFR increased rapidly in low-income countries, from around 10 in the 1970s to over 20 in the 2010s. Meanwhile, SFRs in lower-middle-income countries grew from about 14 in the 1970s to almost 18 in the 2010s. In contrast, upper-middle-income countries and high-income countries experienced only modest increases in SFRs. In particular, the SFR of high-income countries remained between 12 and 14 in the past 50 years, despite the fact that enrollments in higher education have grown significantly.

**Fig. 3:**
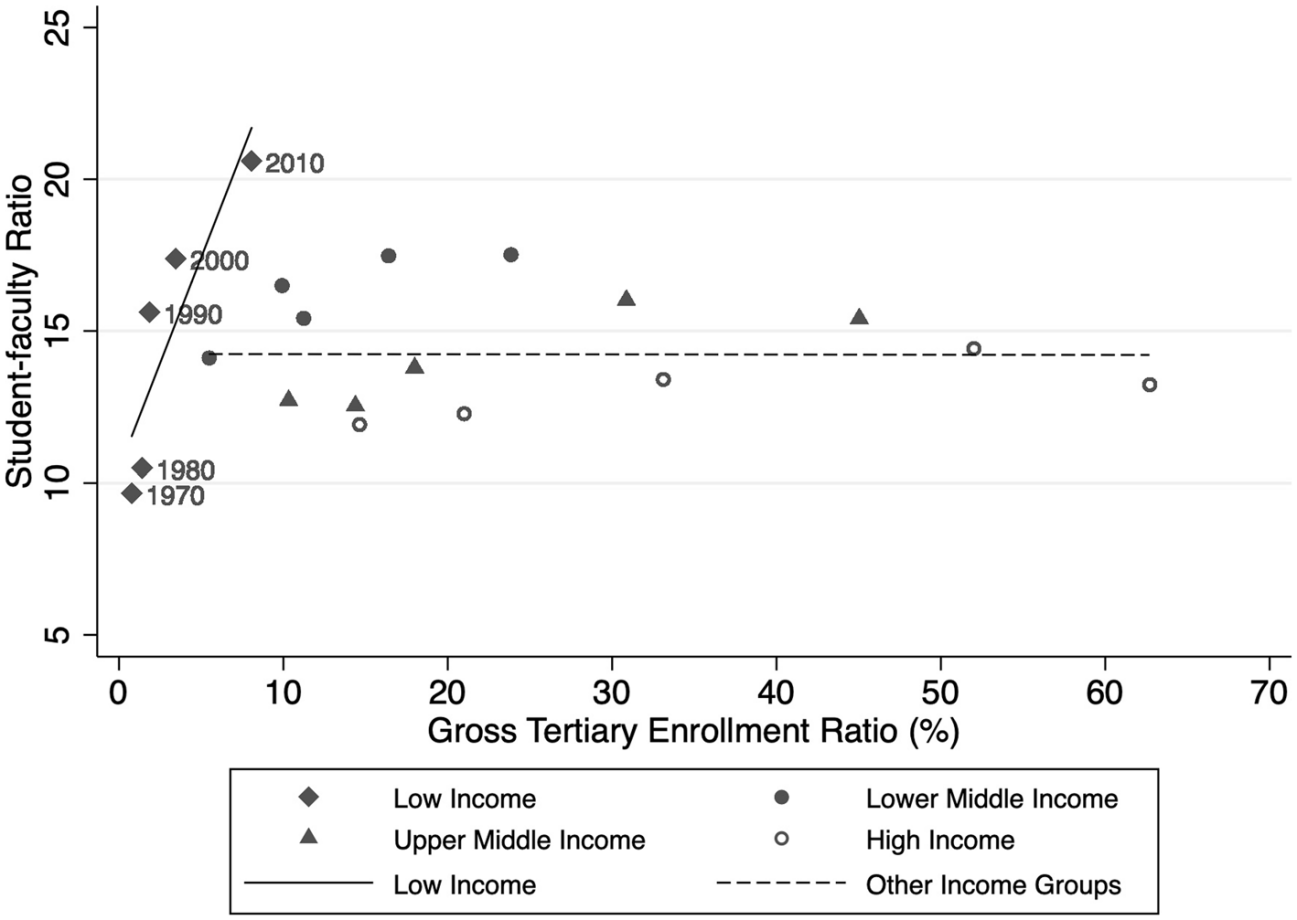
Student-faculty ratios and gross tertiary enrollment ratio, 1970–2018

To examine the relationship between enrollments and SFRs, Fig. 3 shows the bivariate relationship between SFR and GTER, disaggregated by country income group. As the figure clearly shows, higher GTERs are associated with having larger SFRs. Strikingly, this relationship is largest in low-income countries, as compared to other countries, suggesting that SFRs are more responsive to enrollment growth in low-income countries. The figure further suggests that national wealth might be an important factor mediating the impact of massification on SFRs, which we explore further in regression models discussed below.

### Fixed-effect panel regression models, 1970–2018

Table 3 shows the results from the 1970–2018 cross-national data. We include national GDP and spending on tertiary education in model 1 to test the hypothesis that government spending results in more faculty, and therefore, lower SFRs. However, model 1 shows that neither GDP per capita nor tertiary spending are statistically significant.

**Table 3:** Factors predicting student-faculty ratios in higher education, 1970-2018 (N=3067, 166 countries)

	**Model 1**	**Model 2**	**Model 3**	**Model 4**	**Model 5**
GDP per capita (logged)	0.90	0.77	0.87	0.85	0.68
Tertiary Spending (% of GDP)	-0.06	0.19	0.31	0.21	0.19
GTER		0.24***	0.28***		
GTER^2^		-0.00***	-0.00***	-0.00***	-0.00***
Female Faculty (%)			-0.09*	-0.06+	
**GTER x Income Groups**					
Low income				1.27***	1.25***
Lower middle income				0.47***	0.39***
Upper middle income				0.28***	0.33***
High Income				0.25***	0.28***
**Female Faculty x Income Groups**					
Low income					0.05
Lower middle income					0.08
Upper middle income					-0.17*
High Income					-0.13*
Constant	4.78	4.66	5.29	4.63	6.52
N	3067	3067	3067	3067	3067
BIC	17420.17	17200.40	17166.90	16981.21	16933.50

*Note*. Year fixed effect terms are included in all models but are not shown. BIC = Bayesian information criterion; GDP = Gross Domestic Product; GTER = Gross Tertiary Enrollment Ratio.

+ p < 0.1 (two-tailed tests)

* p < 0.05 (two-tailed tests)

** p < 0.01 (two-tailed tests)

*** p < 0.001 (two-tailed tests).

To test the hypothesis that student enrollment in higher education is an important factor driving SFR growth, we add the gross tertiary enrollment rate (GTER) and its squared term in model 2. This allows us to examine a potential non-linear relationship between enrollments and SFRs. The results show that a one percent increase in GTER is associated with 0.24 increase in SFR (*p* < 0.001), controlling for GDP per capita and tertiary spending. This growth, however, plateaus at higher levels of GTER, reflected by the negative coefficient for the square term (*p* < 0.001).

To test the hypothesis that increasing numbers of female faculty slow SFR growth, we add the percentage of female faculty in model 3. The results show that it is associated with lower SFRs. Specifically, one percentage increase in the share of female faculty is associated with 0.09 decrease in SFR (*p* < 0.05).

In our descriptive analysis, we find that SFR growth varies across countries in different income groups, and that low-income countries seem to be driving the growth of SFR worldwide. Therefore, we add the interaction term between GTER and income group in model 4 to examine whether the strength of the relationship between GTER and SFR varies across country income groups. As shown in model 4, low-income countries are most affected by the growth in enrollments. Specifically, a one percent increase in GTER is associated with 1.27 increase in the national SFR in low-income countries, controlling for other factors. In contrast, a one percent increase in GTER is associated with more modest increases in countries of other income groups.

In model 5, we interact the percentage of female faculty with income group to examine if the relationship between female faculty and SFR also varies across country groups. The results show that a one percent increase in female faculty is associated with 0.13 decrease in SFR in high-income countries (*p* < 0.05), and 0.17 decrease in SFR in upper-middle-income countries (*p* < 0.05), controlling for other factors. Meanwhile, the coefficient on female faculty is not significant in low-income and lower-middle-income countries.

Relying on the Bayesian information criterion (BIC) as a measure of model fit, model 5 is the preferred model. This final model shows that GTER is associated with higher SFRs, but also suggests that the relationship between GTER and SFR varies by a country’s overall wealth, and is strongest in low-income countries. The model also shows that a country’s share of female faculty is associated with having lower average SFRs, but this relationship also varies by country wealth. Unlike GTER, the relationship between female faculty and lower SFRs is stronger among high- and middle-income countries than low-income countries, where there is no statistically significant relationship. 

In addition to models shown here, we also examined additional variables that might explain SFRs, including the Gini coefficient, which is a measure of income inequality, and the percentage of enrollment in private higher education. However, neither was statistically significant, and therefore, we do not report on them.

### Fixed-effect panel regression models, 2000–2018

Table 2 shows the summary statistics for the 2000–2018 dataset, which allows to examine a more robust set of independent variables, namely research spending, over a smaller time period and set of countries. In our analytical sample, there are 1218 observations from 107 countries. Table 4 shows the results from the regression models. In addition, we examine if there are any changes in the relationship between GTER and SFR, or between the percentage of female faculty and SFRs in the second dataset.

**Table 4:** Factors predicting student-faculty ratio in higher education, 2000-2018 (N=1218, 107 Countries)

	**Model 6 **	**Model 7 **	**Model 8**	**Model 9**	**Model 10 **
GDP per capita (logged)	1.06	-1.02	-1.04	-1.48	-0.44
Tertiary Spending (% of GDP)	-0.76	-0.57	-0.45	-0.38	-0.65
GTER	0.34***				
GTER^2^	-0.00***	-0.00**	-0.00**	-0.00***	-0.00***
Female Faculty (%)	-0.17*	-0.18**			
R&D Spending (%)	-3.08*	-2.86*	-2.43*	-2.17*	-1.69
**GTER x Income Groups**					
Low income		1.79***	1.49**	1.63**	1.83***
Lower middle income		0.42***	0.42***	0.44***	0.50***
Upper middle income		0.26**	0.27**	0.32***	0.36***
High Income		0.27***	0.29***	0.34***	0.38***
**Female Faculty x Income Groups**					
Low income			0.13	-0.22	-0.23
Lower middle income			-0.15	-0.63*	-0.66*
Upper middle income			-0.20*	-0.77*	-0.79*
High Income			-0.30*	-0.74**	-0.72*
Female Faculty^2^ (%)				0.01+	0.01*
Constant	7.81	25.57*	26.27*	37.71**	26.69
N	1218	1218	1218	1218	1218
BIC	5982.71	5908.88	5910.77	5891.38	5968.13

*Note.* Year fixed effect term is included in Model 10 but is not shown. SFR = Student-faculty Ratio; GDP = Gross Domestic Product; GTER = Gross Tertiary Enrollment Ratio; R&D = Research and Development.

+ p <0.1 (two-tailed tests)

* p < 0.05 (two-tailed tests)

** p < 0.01 (two-tailed tests)

*** p < 0.001 (two-tailed tests).

To test our hypothesis on the relationship between research intensity and SFR, we add a variable for spending on R&D as a percentage of GDP in model 6. Results show that it is negatively associated with SFR; specifically, a 1% increase in R&D spending is associated with a 3.08 decrease in SFR (*p* < 0.05), controlling for other factors. This finding suggests that a country’s research spending is associated with having a higher number of instructors per student. While this relationship may not be causal, the fact that research spending is associated with lower SFRs does seem to indicate that when countries have an orientation towards research, they are also likely to have lower SFRs on average.

In line with our prior findings that the relationship between GTER and SFR varies by country income group, we add an interaction term between GTER and income group in model 7. The results are similar to the findings in the 1970–2018 dataset: SFRs in low-income countries are most affected by the growth in enrollments. A one percentage increase in GTER is associated with 1.79 increase in SFR (*p* < 0.001) in low-income countries, controlling for other factors, compared to a 0.26 increase in upper-middle-income countries (*p* < 0.01) and a increase in high-income countries (*p* < 0.001).

Similarly, we also add the interaction between the percentage of female faculty and income group in model 8. We find that a one percentage increase in female faculty is associated with 0.20 decrease in SFRs in upper-middle-income countries (*p* < 0.05) and a 0.30 decrease in high-income countries (*p* < 0.05), controlling for other factors. As in the prior models, the coefficients for low-income and lower middle-income countries are not significant.

In model 9, we add the square term of female faculty to investigate if the strength of the relationship between female faculty and SFR changes at higher levels of female representation. We find that the coefficient on the square term is positive, indicating that the impact of female faculty on SFRs is moderated at higher levels of female faculty (*p* < 0.10). The model also shows a stronger and statistically significant negative relationship between female share of faculty and SFRs in middle- and high-income countries.

The dataset spans 18 years, a long period of time, and a statistical test in Stata suggests that a year fixed effect might be needed in the model to account for this time effect (*p* < 0.05). Therefore, we add year fixed effect in model 10. However, year fixed effects do not seem to improve model fit. Instead, using BIC as a criterion for model fit, model 9 is our preferred model, as the BIC is the lowest among all the models tested.

**Fig. 4:**
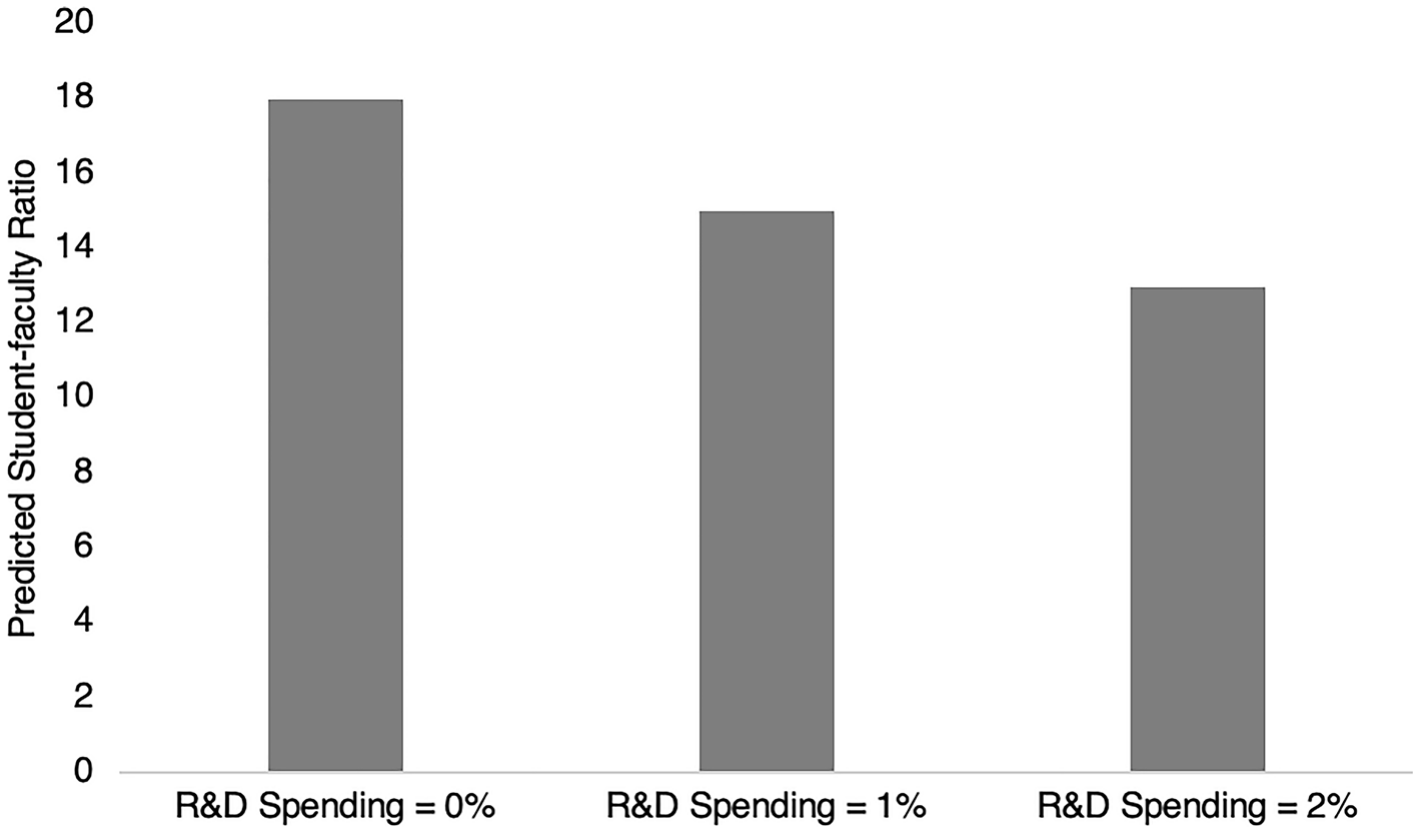
Predicted student-faculty ratio by R&D spending, 2000–2018

In conclusion, our preferred model 9 suggests that in addition to the factors identified in the 1970-2018 dataset, spending on R&D is associated with lower SFRs. Specifically, one percentage increase in R&D spending is associated with 2.17 decrease in SFR (*p* < 0.05), controlling for other factors. To visualize the impact of R&D spending on SFR, we calculate the predicted SFR for three levels of R&D (i.e., 0%, 1% and 2%), which correspond to approximately the first, seventy-fifth and ninetieth percentiles of R&D spending. These are plotted in Figure 4. Increasing R&D spending from 0 to 1% of GDP, keeping other factors the same, is associated with a decrease in national SFR from around 18 to around 15. Meanwhile, spending 2% of GDP on R&D is associated with an estimated national SFR of roughly 13.

In addition, as with the first set of models, we find that the relationship between GTER and SFR varies by country’s wealth. In low-income countries, a one percentage increase in GTER is associated with a 1.63 increase in SFR (*p* < 0.01) compared to only a 0.34 increase in high-income countries, controlling for other factors. A clear finding is that SFRs are more responsive to growing enrollments in low-income countries than anywhere else. 

Interestingly, the relationship between the percentage of female faculty and SFR also varies by country’s wealth. A one percent increase in the female share of faculty is associated with a 0.74 decrease in SFR (*p* < 0.05) in high-income countries, and similar values in middle-income countries, controlling for other variables. However, in low-income countries, a one percent increase in female faculty is associated with a much smaller decrease, and the coefficient is not statistically significant.

**Fig. 5:**
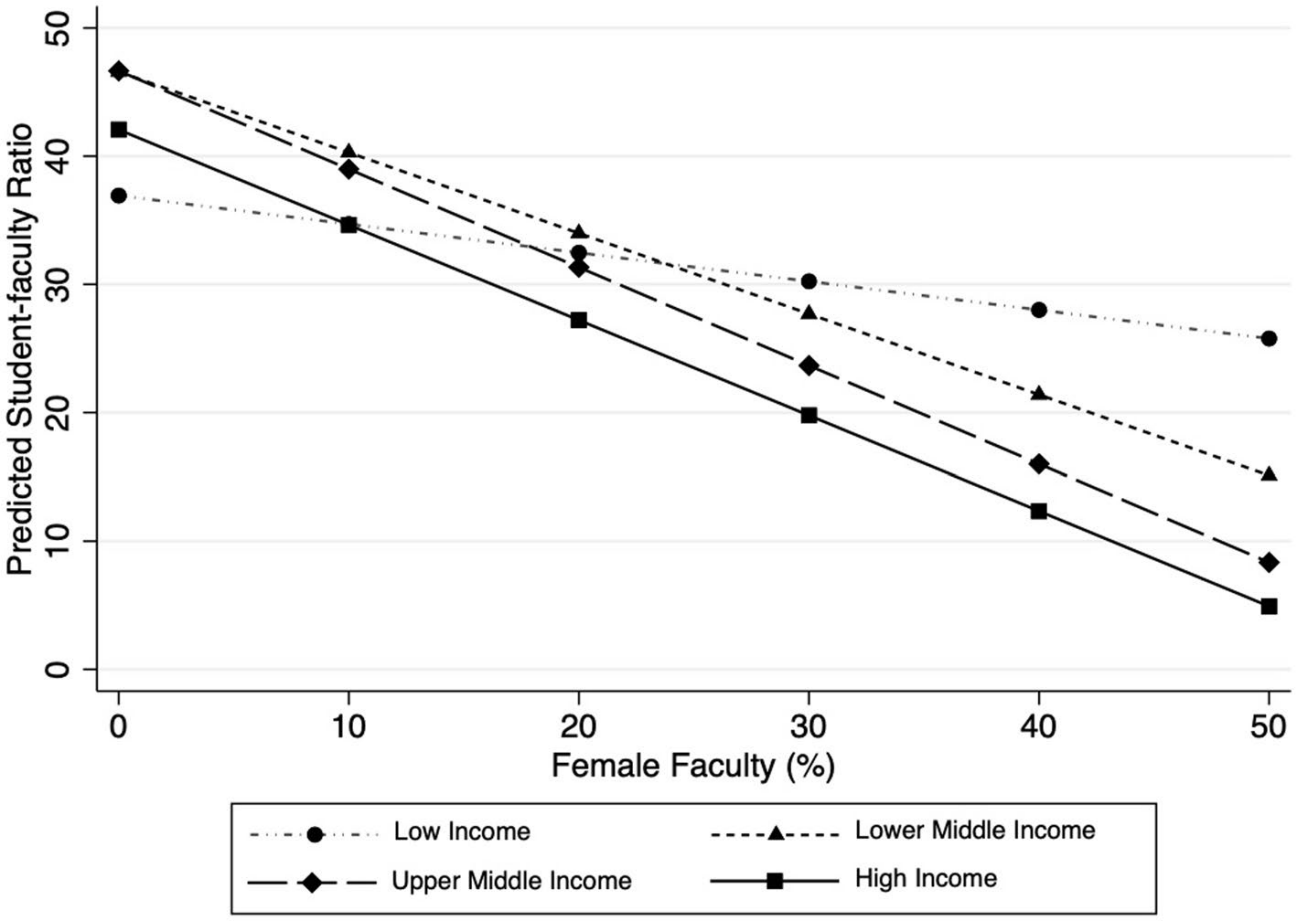
Predicted student-faculty ratio by female faculty and income groups, 2000–2018

To visualize the relationship between female faculty and SFRs across countries, we calculated the predicted SFR at different proportions of female faculty for each country income group, as shown in Fig. 5. The figure shows that the negative relationship between the percentage of female faculty and the SFR is weaker in lower-income countries than other countries, where the strength of the relationship is similar. For example, an increase of female faculty from 10% to 50% is expected to decrease the national SFR from 35 to 26 in low-income countries compared to a much larger decline, from 35 to 5, in high-income countries.

In addition to the variables shown, we tested a number of other variables, including the number of PhD graduates. We hypothesized that the number of PhD graduates is an indicator of the supply of faculty labor and may be associated with lower SFRs. However, this variable is not statistically significant. We also tested whether systemic differentiation, operationalized as the percentage of total enrollments in the vocational tertiary sector (i.e., ISCED 5) played a role. However, this variable is not consistently significant in our models.

Given the large number of missing values, we checked the robustness of our final models by comparing (1) models without missing values filled and (2) models with missing values filled with only interpolated values to our full models. In the 1970–2018 dataset, findings remained consistent using only interpolation (see Table 7 in the Appendix). In the 2000–2018 dataset, findings remained consistent except for the significance level of R&D, the square term of female faculty, and the coefficient for country income group (see Table 8 in the Appendix). However, the direction of the variables remained consistent. Therefore, we consider our model specification robust and report findings with more complete datasets.

## Discussion

This article examines SFRs globally and cross-nationally over time. One of the most striking findings, shown in Fig. 1, is that there has been a marked increase in the average number of students per faculty over the past 50 years, validating the rising concern over massification pressures in higher education. In 1970, the global average SFR was approximately 12, and by 2018, it was 17. Unsurprisingly, rising SFRs seem to be a response to massification pressures—Fig. 3 and our regression analyses show that a country’s gross tertiary enrollment rate is positively and statistically significantly associated with its SFR.

That said, as shown in both Fig. 2 and Fig. 3, we also find significant variation in SFR growth across countries, and more precisely, that these differences are related to a country’s national wealth. In particular, we find that since 1970, SFRs have been growing most rapidly in low-income countries and have also grown substantially in lower-middle-income countries. In contrast, the average SFR in high-income countries has hardly changed since 1970, raising the question: why not? Gross tertiary enrollment rates in high-income countries have increased substantially since the 1970s, with seemingly little impact on the national SFR. This surprising finding suggests that expansion of access does not necessarily result in increases in SFRs. Rather, national-level characteristics moderate the relationship.

In examining what national factors are associated with changes in SFRs, we confirmed the role that certain factors play. First, massification is a major driver of SFR growth. This relationship is particularly large for low-income countries, as depicted in Fig. 3 and confirmed in both sets of regression models. This finding seems to substantiate concerns over quality in low-income countries. In particular, it suggests that low-income countries have thus far been unable to meet expanding enrollments with equivalent faculty hires, at least to the extent that wealthier countries have. We cannot say why this is the case; it may be due to a lack of qualified instructors, a lack of financial resources, less reliance on contract labor, or other factors. This is an area for future research. 

In addition, our findings suggest that a higher percentage of female faculty in a country is associated with lower SFRs. There are many possible causal mechanisms at work, and our regression analyses do not necessarily indicate a direct causal linkage. That said, the literature on women faculty has found a “pipeline” of women that leads from university into faculty. In this literature, scholars have found that enrolling more women students leads to a growing number of women entering the academic profession (Wotipka et al. 2018). A country’s percentage of female faculty is positively associated with it having more PhD graduates overall, and in this sense, may be a proxy for an oversupply of graduates, or an availability of contract labor. However, we also cannot rule out a more direct causal link: female faculty, who tend to be younger on average and may face pay gaps, may reduce a country’s national SFR more directly by allowing a country to hire more faculty overall. This is an important area for future research, particularly in countries where the national SFR is being used as an indicator of quality or capacity. However, our regression models suggest that the relationship between female faculty and lower SFRs is weak in low-income countries. The reasons behind this are unclear and future research is needed to explore this relationship.

Another important finding from the second set of regression models is that national spending on research is negatively associated with national SFR. This confirms our hypothesis that increasing national research intensity may be associated with lower overall SFRs, in line with the findings on institutional SFRs. There are many possible mechanisms at work. As national governments rely on higher education to advance research, primarily conducted by faculty, spending on research may mean that countries are able to increase the total number of faculty in the system. This might also mean that countries also have to balance research and teaching functions of higher education by hiring more teaching faculty.

However, the other factors we hypothesized to matter, including GDP per capita and tertiary spending were not statistically significantly associated with SFRs, nor were a number of other factors we tried, including the private share of enrollments, total number of PhD graduates, or systemic differentiation. The lack of significance may be due to limitations with the data; therefore, as more data becomes available, future research can investigate the role of these factors.

**Fig. 6:**
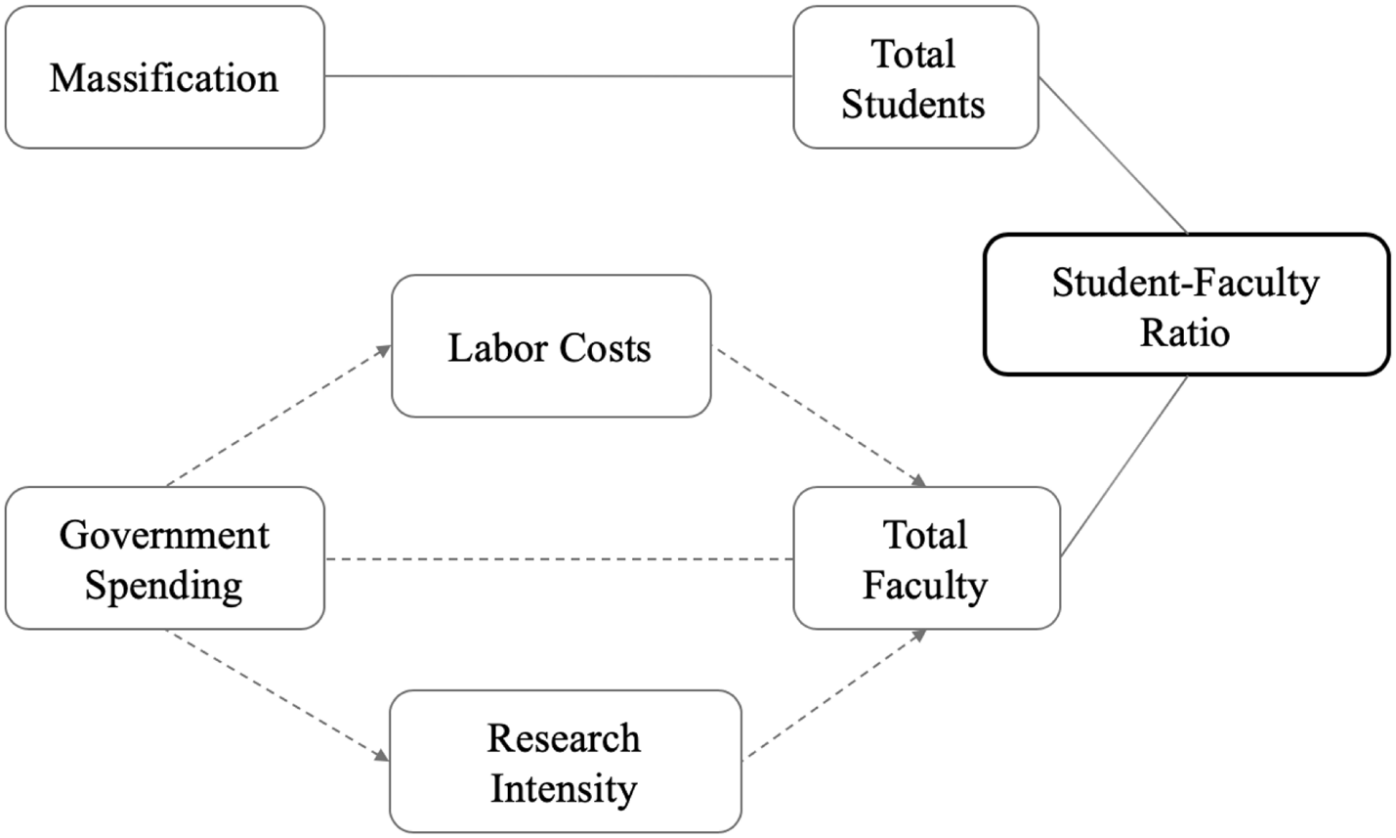
Conceptual framework for national student-faculty ratios in higher education

Building on our findings, in Fig. 6, we propose a conceptual framework to understand national SFRs. The figure is based on the factors shown to be statistically significantly related to SFRs in our various regression models, namely enrollment rates, research intensity, and labor costs. We use solid lines to indicate the relationship between massification, enrollments, and students to indicate robust findings where causal mechanisms are clear. In contrast, we use dotted lines to indicate the fact that causal mechanisms concerning factors associated with total faculty are less understood. Future research can build on this framework to further explore how other factors also influence SFRs. Of particular need is a better way to measure faculty labor costs and the percentage of faculty on contract.

## Conclusion

This article investigates national SFRs over the past five decades and explanations for growth. Although we acknowledge that the SFR is a narrow indicator and does not necessarily imply student learning, it may serve as a cross-nationally comparable indicator of aggregate system capacity. Moreover, given the usage of SFRs as an indicator of quality in educational planning, this article has developed an initial conceptual framework to examine factors associated with national SFRs. Centered on the quantity versus quality dilemma in higher education, our conceptual framework shows that maintaining “quality” in a higher education system is a dynamic interaction between student enrollment and faculty labor.

Moreover, we find that national SFRs have been growing worldwide over the past five decades, validating the concerns of many scholars and policymakers in comparative higher education. However, in a surprising finding, we also find that this growth does not appear to be unchecked. Our models suggest that high-income countries have been able to maintain stable SFRs even though their systems have expanded significantly. In contrast, low-income countries have seen substantial growth in SFRs, albeit from very low ratios in the 1970s. That said, in line with the global trend that growth is not indefinite, the growth in SFRs in low-income countries may end up levelling off, reflecting trends in other parts of the world.

Our models suggest that there are ways to maintain relatively low national SFRs and that role differentiation of faculty labor, with more research-intensive faculty and more female faculty both parts of the equation. However, more work is needed to better understand and untangle these relationships and the causal mechanisms at work, particularly examining why the percent of female faculty is related to lower SFRs, and why this relationship is weaker in low-income countries.

Additionally, future research must examine the role of contract faculty labor on national SFRs, as the “adjunctification” of faculty labor is a major trend in many countries. Relatedly, future research can examine the impact of differentiation within and between higher education institutions on national SFRs. We might ask how SFRs vary based on where (e.g., private versus public, or teaching-focused versus research-focused institutions) and in which disciplines (science, technology and engineering programs versus social science and humanities) students are disproportionally enrolled. Finally, while our analysis has focused on drivers of the national SFR, in future research, scholars must explore how national SFRs are related to other important outcomes, such as national graduation rates, publication rates, or other aspects of system-wide quality. Answering these questions will provide important insight on the changing landscape of higher education, particularly student and faculty dynamics in an era characterized by concerns over the trade-off between quantity and quality in higher education.

## Appendix

### Missing values

(see tables 5 and 6)

**Table 5:** Total number of observations filled in the 1970–2018 dataset

**Variable **	**Total**	**Number of Missing Values Filled**
SFR	3067	0
GDP per capita (logged)	3067	0
Tertiary Spending (% of GDP)	3067	1480
GTER	3067	27
Female Faculty	3067	699

**Table 6:** Total number of observations after filling in missing and the number of missing values filled in the 2000–2018 dataset

**Variable Name**	**N**	**Number of Missing Values Filled**
SFR	1218	0
GDP per capita (logged)	1218	0
Tertiary Spending (% of GDP)	1218	464
GTER	1218	4
Female Faculty	1218	75
R&D Spending (% of GDP)	1218	352

### Robustness check

(see tables 7 and 8)

**Table 7:** Factors predicting student-faculty ratio, 1970–2018

	Model A1	Model A2	Model A3
GDP per capita (logged)	0.20	0.83	0.68
Tertiary spending (% of GDP)	0.55	0.49	0.19
GTER × income groups			
Low income	0.75	1.19**	1.25***
Lower middle income	0.29**	0.37***	0.39***
Upper middle income	0.19*	0.29***	0.33***
High income	0.17*	0.24**	0.28***
GTER^2^	− 0.00+	− 0.00**	− 0.00***
Female faculty × income groups			
Low income	0.29*	0.15	0.05
Lower middle income	0.06	0.03	0.08
Upper middle income	− 0.05	− 0.18*	− 0.17*
High income	− 0.17*	− 0.16*	− 0.13*
Constant	15.60	8.78	6.52
N	1259.00	2249.00	3067.00
BIC	6732.57	12352.66	16933.55

Model A1: no missing values filled in; model A2: missing values filled in with interpolation only; model A3: missing filled with interpolation and Cohen and Cohen country means; year fixed-effect terms are included in all models but are not shown

*BIC* Bayesian information criterion, *GDP* gross domestic product, *GTER* gross tertiary enrollment ratio

**p* < 0.05 (two-tailed tests); ***p* < 0.01 (two-tailed tests); ****p* < 0.001 (two-tailed tests)

**Table 8:** Factors predicting student-faculty ratio, 2000–2018

	Model A4	Model A5	Model A6
GDP per capita (logged)	− 2.01	− 2.42+	− 1.48
Tertiary spending (% of GDP)	− 0.05	− 0.67	− 0.38
Female faculty^2^ (%)	0.00	0.01**	0.01+
GTER × income groups			
High income	0.41***	0.48***	0.34***
Low income	1.40+	0.48	1.63**
Lower middle income	0.40**	0.55***	0.44***
Upper middle income	0.35**	0.46***	0.32***
GTER2	− 0.00**	− 0.00**	− 0.00***
R&D spending (% of GDP)	− 2.92+	− 2.93*	− 2.17*
Female faculty × income groups			
High income	− 0.70*	− 1.20***	− 0.74**
Low income	− 0.19	− 0.67*	− 0.22
Lower middle income	− 0.41	− 1.33**	− 0.63*
Upper middle income	− 0.55	− 1.33**	− 0.77*
Constant	40.05**	56.97***	37.71**
N	712.00	725.00	1218.00
BIC	3265.54	3259.41	5891.38

Model A4: no missing filled; model A5: missing filled with interpolation; model A6: missing filled with interpolation and Cohen and Cohen country means

*BIC* Bayesian information criterion, *SFR* student-faculty ratio, *GDP* gross domestic product, *GTER* gross tertiary enrollment ratio, *R&D* research and development

^+^*p* < 0.1 (two-tailed tests); **p* < 0.05 (two-tailed tests); ***p* < 0.01 (two-tailed tests); ****p* < 0.001 (two-tailed tests)

## References

[CR1] Aksnes DW, Sivertsen G, van Leeuwen TN, Wendt KK (2017). Measuring the productivity of national R&D systems: Challenges in cross-national comparisons of R&D input and publication output indicators. Science and Public Policy.

[CR2] Altbach PG (1999). The logic of mass higher education. Tertiary Education & Management.

[CR3] Altbach PG, Pacheco IF (2012). Paying the professoriate: A global comparison of compensation and contracts.

[CR4] Altbach PG, Reisberg L, Rumbley LE (2009). Trends in global higher education: Tracking an academic revolution.

[CR5] Auranen O, Nieminen M (2010). University research funding and publication performance—An international comparison. Research Policy.

[CR6] Baker D (2014). The schooled society: The educational transformation of global culture.

[CR7] Barakat B, Shields R (2019). Just another level? Comparing quantitative patterns of global expansion of school and higher education attainment. Demography.

[CR8] Card, D., & Krueger, A. B. (1996). Labor market effects of school quality: Theory and evidence. NBER Working Paper Series. Working Paper 5450. Massachusetts, US: National Bureau of Economic Research.

[CR9] Carnoy M, Loyalka P, Dobryakova M, Dossani R, Kuhns K, Wang R (2013). University expansion in a changing global economy: Triumph of the BRICs?.

[CR10] Chang D-F, Nyeu F-Y, Chang H-C (2015). Balancing quality and quantity to build research universities in Taiwan. Higher Education.

[CR11] Chang D-F, Wu C, Ching GS, Tang C (2009). An evaluation of the dynamics of the plan to develop first-class universities and top-level research centers in Taiwan. Asia Pacific Education Review.

[CR12] Cohen J, Cohen P, West SG, Aiken LS (2003). Applied multiple regression/correlation analysis for the behavioral sciences.

[CR13] Davies B, Bansel P (2005). The time of their lives? Academic workers in neoliberal time (s). Health Sociology Review.

[CR14] Ehrenberg, R. G., Rizzo, M.J., & Jakubson, G.H. (2003). Who bears the growing cost of science at universities? CHERI Working Paper #35. New York, US: Cornell University ILR School.

[CR15] Finkelstein, M. J. (2012). *The power of institutional and disciplinary markets, in Paying the professoriate: A global comparison of compensation and contracts, *(Eds.) Altbach, Philip, Liz Reisberg, Maria Yudkevich, Gregory Androushchak and Ivan Pacheco. New York, US: Routledge, 318–328.

[CR16] Finkelstein MJ, Jones GA (2019). Professorial Pathways: Academic Careers in a Global Perspective.

[CR17] Geuna A, Martin BR (2003). University research evaluation and funding: An international comparison. Minerva.

[CR18] Goenner CF, Snaith SM (2004). Predicting graduation rates: An analysis of student and institutional factors at doctoral universities. Journal of College Student Retention: Research, Theory & Practice.

[CR19] Guri-Rosenblit S, Šebková H, Teichler U (2007). Massification and diversity of higher education systems: Interplay of complex dimensions. Higher Education Policy.

[CR20] Harber C (2014). Education and international development: Theory, practice and issues.

[CR21] Hayward FM (2017). Lessons learned from strategic planning for improved teaching and learning in developing economies. Planning for Higher Education.

[CR22] Hazelkorn E (2015). Rankings and the reshaping of higher education: The battle for world-class excellence.

[CR23] Hornsby DJ, Osman R (2014). Massification in higher education: Large classes and student learning. Higher Education.

[CR24] Konstantopoulos S, Chung V (2009). What are the long-term effects of small classes on the achievement gap? Evidence from the lasting benefits study. American Journal of Education.

[CR25] Konstantopoulos S, Li W (2012). Are there additional benefits from being in small classes for more than one year?. Educational Research and Evaluation.

[CR26] Marginson S (2016). The worldwide trend to high participation higher education: Dynamics of social stratification in inclusive systems. Higher Education.

[CR27] Mason MA, Goulden M (2004). Marriage and baby blues: Redefining gender equity in the academy. The Annals of the American Academy of Political and Social Science.

[CR28] Mohamedbhai G (2014). Massification in higher education institutions in Africa: Causes, consequences and responses. International Journal of African Higher Education.

[CR29] Mok K-H (2003). Similar trends, diverse agendas: Higher education reforms in East Asia. Globalisation, Societies and Education.

[CR30] Paananen M (2020). Fluctuating child–staff ratio: governing by numbers in Finnish early childhood education. International Studies in Sociology of Education.

[CR31] Renzulli LA, Reynolds J, Kelly K, Grant L (2013). Pathways to gender inequality in faculty pay: The impact of institution, academic division, and rank. Research in Social Stratification and Mobility.

[CR32] Saint W (2004). Higher education in Ethiopia: The vision and its challenges. Journal of Higher Education in Africa/Revue de l’enseignement Supérieur En Afrique.

[CR33] Schenker-Wicki A, Inauen M (2012). The economics of teaching. Higher Education Management and Policy.

[CR34] Schofer E, Meyer J (2005). The worldwide expansion of higher education in the twentieth century. American Sociological Review.

[CR35] Scott P (1995). The meanings of mass higher education.

[CR36] Teichler U (1998). Massification: A challenge for institutions of higher education. Tertiary Education and Management.

[CR37] Tight M (2019). Mass higher education and massification. Higher Education Policy.

[CR38] Toutkoushian RK, Smart JC (2001). Do institutional characteristics affect student gains from college?. The Review of Higher Education.

[CR39] Trow M (1972). The expansion and transformation of higher education. International Review of Education.

[CR40] Trow M (2007). Reflections on the transition from elite to mass to universal access: Forms and phases of higher education in modern societies since WWII. International handbook of higher education.

[CR41] UNESCO, United Nations Education Science and Cultural Organization. (2020). *Educational Statistics*. Montreal, Canada: UNESCO Institute of Statistics. Available at: https://data.uis.unesco.org/

[CR42] United Nations Educational Scientific and Cultural Organization (2010). Education for All Global Monitoring Report, 2010. Reaching the Marginalized.

[CR43] Watson K, Handal B, Maher M, McGinty E (2013). Globalising the class size debate: myths and realities. Journal of International and Comparative Education (JICE).

[CR44] Welch A (2012). Academic salaries, massification and the rise of an underclass in Australia.

[CR45] Wotipka CM, Nakagawa M, Svec J (2018). Global linkages, the higher education pipeline, and national contexts: The worldwide growth of women faculty, 1970–2012. International Journal of Comparative Sociology.

[CR46] Yang L, McCall B (2014). World education finance policies and higher education access: A statistical analysis of World Development Indicators for 86 countries. International Journal of Educational Development.

